# Police of the psyche: the psychiatrist and psychiatry in Spanish punk songs

**DOI:** 10.1192/bjb.2023.47

**Published:** 2024-06

**Authors:** Fabian Pavez, Erika Saura, Pedro Marset

**Affiliations:** 1 Mental Health Centre of Lorca, Lorca, Spain; 2 Association of Relatives and People with Mental Illness in Águilas and the Surrounding Area (AFEMAC), Aguilas, Spain; 3 University of Murcia, Murcia, Spain

**Keywords:** Thematic analysis, representation, psychiatry, stereotype, popular music

## Abstract

Previous research has revealed that stigma is not restricted to people with mental health problems but extends to the professionals involved in their care and treatment. Unlike other artistic manifestations, the study of the depictions of psychiatry in popular music is still a less-explored topic. This article addresses the subcultural portrayals of the psychiatrist and psychiatric treatments within Spanish popular music. The predominance of negative depictions of mental health professionals as social control agents was a striking finding, given the topicality that characterises punk music. It is suggested that the allegorical role assigned to the psychiatrist in such a specific narrative framework, marked by ideological factors, could potentially explain these findings. In contrast to other cultural manifestations that show a tendency towards more balanced views of psychiatric treatment and practice, the negative representations in Spanish punk songs seem to have evolved little over decades, reflecting outdated views of the psychiatric approach.

‘Science's claw draws the boundary between my freedom and its abyss.

The architects of the mind build prisons.

Adaptation or extinction is their psychotherapy.

Insanity is just another pretext to ensure social control.

I feel the light dancing for me. You only see the darkness.’

‘The secret of vampires’ [‘El secreto de los vampiros’] (Ruido de Rabia, 1993)

Previous research has revealed that stigma is not restricted to people with mental health problems but extends to the professionals involved in their care and treatment,^1–9^ in what Goffman describes as ‘courtesy stigma’ or ‘stigma by association’,^10^ i.e. the stigmatisation of a person as belonging to the same social group as a discredited individual. This stigma is reinforced by cultural descriptions of professionals and patients.^2^

Research on various cultural products (film and television, literature, comics, video games, internet videos and other entertainment media) suggests the predominance of negative portrayals of mental health professionals and treatments.^1–17^ It leads to a gap in the knowledge and attitudes of the general population. For many people (particularly the young), these depictions of psychiatrists and their patients may be their first encounters with psychiatric disorders and treatment.^2^

Despite the ubiquity of popular music in everyday life,^18^ little attention has been paid to representations of mental health practices in music. In contrast, research on cinema has been particularly prolific in this area. However, the vast majority of film studies fall into the category of textual analysis, i.e. the use of informal interpretative methods.^19^

As far as we are aware, the only study of popular song lyrics in this area involved a critical discourse analysis of 24 pieces, leading to the identification of three broad themes: ‘banal therapy’ (songs that name therapy without being explicitly oriented towards a representation of it), ‘the non-therapeutic relationship’ (i.e. the absence of a genuine therapeutic bond, including financial exploitation, deception, inauthenticity, omniscient proclamation and medicalisation) and ‘I know, therefore I can’ (resistance to therapy as an oppressive tool of discipline and control).^20^

With these elements in mind, this article addresses the subcultural portrayals of the psychiatrist and psychiatric treatments within Spanish punk music.

## Method

We conducted a thematic analysis of the lyrics of Spanish punk songs recorded between 1981 and 2010. Thematic analysis is a systematic method for identifying, organising and understanding the patterns of meaning present in a data-set.^21^ It was considered appropriate for our objectives because of its accessibility, flexibility, systematisation and replicability.

After a purposive sampling guided by bibliographic, documentary and web sources (listed in the Supplementary Material, available online at https://doi.org/10.1192/bjb.2023.47), 5647 songs were listened to, identifying and transcribing all songs with psychosis-related content as the main lyrical theme. Psychotic disorders were chosen because of their paradigmatic character among mental disorders. Although each song was considered the main context unit, they could contain one or more themes of interest. The concept of ‘reference’ alludes to the song phrases with contents related to our scope. At a quantitative level, duplicated references (e.g. chorus or refrain) were counted once.

The structure proposed by Braun & Clarke^21^ was used for a thematic analysis in six phases of psychiatry-related themes. The participation of two independent coders aimed at optimising internal consistency.

### Ethics

This non-interventional study used public data without identifiable personal data and therefore did not require ethics approval.

## Results

### Thematic clusters

Psychosis-related content as the main theme (including symptoms, syndromes or psychiatric diagnoses related to psychosis) was identified in 1.4% of songs (79 songs; 185 references).

Thematic analysis of these 79 songs revealed three main clusters of themes: clinical-therapeutical (39.46%; 73 references), social dimension of psychosis (31.35%; 58 references) and a mixed group (22.7%; *n* = 42 references) combining themes from the two previous clusters.

The most frequent references to clinical-therapeutical issues included allusions to symptoms (*n* = 31 references), treatments (*n* = 16), hospital admission (*n* = 8) and substance use/misuse (*n* = 8).

Treatments and psychiatric hospital admission intersected clinical-therapeutical and social aspects. Notably, in 60 songs describing psychiatric syndromes or diagnoses as the main theme, about one-third (*n* = 19 songs) referred to treatments and admission, showing a positive picture in only one case. The remainder were neutral (*n* = 4) or negative (*n* = 14).

### The psychiatrist

Portrayed at best as a well-meaning but incompetent character (in other songs, as sadistic, ‘mad’, an enemy, manipulative, controlling or ‘police of the psyche’), depictions of psychiatrists were extremely negative. There were no precise distinctions, and in some cases, the figure of the psychiatrist, the surgeon, the scientist (usually the stereotype of the mad or evil scientist) and the psychologist were conflated.

Representations of the psychiatrist invariably appeared linked to those of psychiatric hospital admission (mainly of the ‘madhouse’ type). In this particular narrative framework, mental health professionals are usually depicted as a complement to ‘the madman’ (in opposition to them) through a negative stereotype showing the psychiatrists as agents of social control.

Another relevant aspect was the absence of depictions of female therapists. Only one secondary allusion to a female mental health nurse was found.

### Treatments

Treatments were mentioned marginally and, in general, they complemented the image of the psychiatrist as an agent of repression. The descriptions were superficial, usually stereotyped, without delving into the treatments’ characteristics, techniques or effects. For example, terms such as ‘drugging’ or ‘poisoning’ were used to refer to psychopharmacotherapy.

In one song (‘The psychiatric ward’ [El Psiquiátrico], Brote Sikótico, 2007), the pharmacological treatment described as ‘poisoning’ appeared as a protection against the individual's delusions. However, the categorisation as ‘poison’ reveals the harmful nature attributed to psychiatric medications at the somatic level. Additionally, the idea of ‘drugging him to protect him’ suggests a paternalistic approach where the dynamics of power and submission are implicit.

Although mistrust of pharmacological treatment and the psychiatrist is emphasised, the jocular song ‘Prozak, Etumina y Haloperidol’ (Manolo Kabezabolo, 2007) provides a counterpoint by advocating (rather ironically) illness awareness and therapeutic adherence: ‘I like girls who by conviction take treatment and medication: injectables and pills, Rubifen, Rohypnol, Prozac, Etumine and haloperidol … decontrol!’. In this case, the idea of ‘decontrol’ subverts the cultural meaning of psychopharmacology as an instrument for behavioural ‘control’. By appropriating the pharmacotherapeutic arsenal for a contrary purpose, it challenges the role of medication within the hegemonic culture. In a broad sense, this song reaffirms the otherness of punk music, sealing its identification with ‘madness’ as a general way of understanding mental illness (fundamentally characterised by the ‘lack of control’). Likewise, it expresses the speaker's inclination towards people who are different, those who do not fit into the mainstream social pattern.

As for non-pharmacological treatments, references to electroconvulsive therapy, psychosurgery, psychotherapies, physical restraint and ‘alternative solutions’ were found (Table 1).

**Table 1:** Non-pharmacological treatments or approaches as described in 79 Spanish punk songs with psychotic disorders or symptoms as the main theme (1981–2010)^a^

Approaches or treatments	Examples (verbatim, from the Spanish)
Electroconvulsive therapy	‘Up to my balls with my mind and the people who live inside me [ … ] I am, and have been, so many men that the daily menu is *electricity*’ (‘Whom am I being?’ [¿Quién vengo siendo?], Siniestro Total, 1997)
Psychosurgery	‘Lucid *chainsaws opening up a broken path towards my brains* that now have to jump so as not to pop’ (‘The madman’ [El Loco], El corazón del sapo, 1998)
Psychotherapies	‘They are not two. She is just one; it is a case of split personality. I have already taken her to be *psychoanalysed*’ (‘2 Loves’ [2 Amores], Los vegetales, 1996)
Physical restraint	‘*Tied to the bed* once again, you think about how you can escape from your anguish and anxiety, and this damned place’ (‘The Psychiatric Ward’ [El Psiquiátrico], Brote Sikótiko, 2007) ‘At good hours, *long sleeves that are tied behind your back* … ’ (‘Whom am I being?’ [¿Quién vengo siendo?], Siniestro Total, 1997)
Other physical methods	‘*Cold showers* can help you to get rid of that discomfort. Schizophrenia is on the rise, I hear crying and wailing … ’ (‘Schizophrenia’ [Eskizofrenia], Habrá Kadáver, 2001)
Other attempted solutions / Inefficacy	‘I want to silence those million voices that sound in the dark; they do not let me rest. *Witchcraft, Satanism, acupuncture, occultism*, I have tried, *nobody can silence them*’ (‘1.000.000 voices’ [1.000.000 de voces], Commando 9mm, 2001)

a. Italics added.

The two references to electroconvulsive therapy were mainly caricatural, without any allusion to its effectiveness or its role as a medically acceptable treatment. In one case, this was in a humorous context and in the other, as a form of punishment. Psychosurgery appeared in only one case, understood as a further means of control and subjugation of ‘the madman’. Psychotherapy was described similarly: ‘adaptation or extinction is their psychotherapy, insanity is just another pretext to ensure social control’ (‘The secret of vampires’ [El secreto de los vampiros], Ruido de Rabia, 1993).

Another piece depicted psychoanalysis as a possible ‘cure’ for ‘schizophrenia’ (‘2 Loves' [2 Amores], Los vegetales, 1996). Strikingly, it places psychoanalysis as an in-patient treatment and confuses dissociative identity disorder with schizophrenia. Although it is a light-hearted song, the inaccuracies of the artist's descriptions divert the audience from the reality of psychotic disorders.

The futility of treatments and the trivialisation of psychotic disorders (in terms of minimising their severity) were identified in the assumptions suggesting an individual could recover of their own will: ‘no one can help you, no one can advise you. Wake up from that lie [ … ] Forget that fear that rots your life, wake up from that lie or live behind your bars’ (‘Prisoner of Madness’ [Prisionero de la locura], Subterranean Kids, 1988). Thus, the self-healing and self-will rhetoric (in the song, ‘forget the fear’ and ‘wake up from the lie’) discourages therapy (‘no one can help you, no one can advise you’). In this example, the performer tries to persuade a third party to find a path out. However, this path is presented as an individual's own choice, suggesting absolute knowledge on the part of the artist, who condemns to continued living ‘behind bars’ as ‘prisoner of madness’ those who do not make the decision (as if it depended on their will) to ‘wake up from the lie’.

### Psychiatric hospital admission

Psychiatric in-patient care was depicted as the loss of freedom in a repressive context marked by confinement and seclusion, isolation, coercion, restrictions, discomfort and suffering (Table 2), and the abuse of power by the professionals in charge.

**Table 2:** Depictions of psychiatric hospital admission in 79 Spanish punk songs with psychotic disorders as the main theme (1981–2010)^a^

Views of psychiatric hospital admission	Examples (verbatim, from the Spanish)
Admission as confinement and loss of freedom	‘Thomas is *locked up* in a hospital. They accuse him of being mad because of his imaginary dog’ (‘Thomas’ [Tomás], Trastienda RC, 2006) ‘Some time ago, I was *locked up* in a mental hospital, I can't stand it any longer’ (‘The madman’ [El loco], La Broma de Ssatán, 1982) ‘I'm *locked up* in four walls, surrounded by zombies [ … ] It's medicine's fault’ (‘The madness’ [La locura], Piorreah, 1984)
Admission, discomfort and suffering	‘Science ghettos for sane minds [ … ] Tension, pain, white corridors in white silence, icy and sinister’ (‘The madman’ [El loco], El corazón del sapo, 1998)
Admission and social control	‘The architects of the mind build prisons [ … ] Insanity is just another pretext to ensure social control’ (‘The secret of vampires’ [El secreto de los vampiros], Ruido de Rabia, 1993)
Admission as control of dangerousness	‘They were to blame. Why did they let me go? I want to go back to the psychiatric ward [ … ] And it happened, in a pool of blood it ended up … ’ (‘Inside me’ [Dentro de mí], Cerebros Exprimidos, 1990)

a. Italics added.

Closely related to these ideas, the role of the psychiatric hospital as a tool of social control included the management of the alleged social dangerousness of the mentally ill.

Again, the futility of psychiatric approaches is an idea that seems to be repeated. Some examples of this topic include the inability of psychiatrists to ‘cure’ an individual of his homicidal tendencies (with the consequent recidivism) or the limitations of clinical judgement leading to underestimating such a risk. So, in this kind of narrative, admission becomes ineffective in controlling symptoms, and professionals appear unable to detect that symptoms have not subsided.

## Discussion

Walter suggests that the stigma attached to psychiatrists arguably functions as a way by which society selects those professionals who can ‘comfortably embrace the stigma of their patients’ (p. 550).^6^ This assertion would be plausible for ‘courtesy stigma’^10^ or views of the psychiatrist as ‘mad’, but not necessarily for representations such as those identified in Spanish punk, where the depictions of mental health professionals frequently seem to be placed in opposition to their patients.

In the songs analysed, the representation of the psychiatrist as the depository of projected madness (in other words, the stereotype of the ‘mad psychiatrist’) was considered irrelevant as there were too few examples found. Most depictions assumed madness as part of a subversive identity construction without projecting it onto the psychiatrist, who, on the contrary, appeared as the embodiment of a repressive social system.

The psychiatrist in Spanish punk songs functioned as an allegory of the State as a more abstract entity and, more universally, of the oppression as opposed to the freedom embodied in ‘the madman’, revealing the ideological undercurrents of punk subculture.

The more explicit depictions of the psychiatrist as a repressive agent or, to borrow a phrase from one of the songs, ‘police of the psyche’ (‘Sane’ [Cuerda], Asto Pituak, 2007), appeared as echoes of ideas akin to anti-psychiatry or in the context of an anarchist-inspired sociopolitical philosophy. Implicitly, the psychiatrist appears as an enemy, and treatments take on a negative cast since, in punk music, the identification with ‘the madman’ as ‘the other’ assumes madness as freedom, an alternative way of life, opposition to the social order, the absence of rules or a desired state.

Although relevant in the representations of the psychiatrist in Spanish punk, the stereotype of the repressive agent of society is not the only one present in popular culture, as can be deduced from previous studies,^3,4,8,22^ and it differs from other cultural manifestations that show a tendency towards more balanced views of psychiatric treatment and practice.^2,15,23^

On the other hand, the absence of female therapists in depictions of Spanish punk contrasts with the few but well-differentiated cinematic representations.^3,8^

The limited usefulness of psychiatric treatment was an underlying theme in the lyrics of the identified Spanish punk songs. These findings are consistent with previous studies of cartoons and film.^7–9^

Unlike the descriptions in films, medications appeared more apparent in the songs under analysis, which may be related to the periods of study (the classic film studies were carried out in the late 1980s and early 1990s). However, it is important to note that, although often as a subtext, psychiatric drugs are beginning to gain prominence in the narratives of relatively recent popular cultural productions.^23^

The psychiatric hospital in the songs under analysis remains predominantly outdated, just as in films.^17^ By highlighting its carceral character and frequently exhibited as a ‘madhouse’, the hospital appeared as one more materialisation of psychiatric power. Thus, psychiatrists, hospitals and treatments serve the same purpose: the restriction of ‘the madman's’ freedom, his confinement and separation from the rest of society, and the neutralisation of a threat to the social order.

### Ideology and allegorical function in punk representations of the psychiatrist

The role of the psychiatrist as a metaphor for power and control seems functional to the messages of punk music, which also explains, to some degree, the homogeneity and stability of the representations.

The identified Spanish punk songs were marked by their immutability, despite the course taken by psychiatry between 1981 and 2010. In other words, psychiatry and psychiatric treatments in Spanish punk lyrics were marked by outdated descriptions of an asylum psychiatry, closest to the Francoism era than later models of care. Thus, the Spanish psychiatric reform from 1985 onwards and the new discourses on psychotropic drugs in the 1990s went practically unnoticed in the songs under study.

The monolithic quality of the Spanish punk depictions of the psychiatrist was a striking finding, considering the topicality of punk songs. Song format constraints, intertextual influences and, perhaps more importantly, the relationship with the allegorical function of the psychiatrist's figure in lyrical discourses of Spanish punk may explain these findings.

### Limitations of the study

The findings do not allow the establishment of directionality in the associations, nor is it possible to infer the impact or influence of these representations on audiences or the factors that influence their genesis.

## Conclusions

In the dichotomous cultural construction ‘mentally ill person–psychiatrist’, the allegorical function assigned to each one led to a loss of nuances, sustaining a Manichean vision of good and evil people, victims and tormentors, persecuted and persecutors. In this picture, the psychiatrist appeared as an agent of repression, reserving the victim role for ‘the madman’.

In Spanish punk lyrics, medicines ‘drug’ people and are harmful, hospitals appear as centres of seclusion, as a hell of long corridors devoid of humanity, and psychiatrists are agents who, through coercion and questionable treatments, force the individual to fit into a normality mould. The patient's opinion is not heard; the mentally ill are subjugated, frightened, confined, restricted and harmed; their individuality is annulled. They do not appear as someone to whom professionals dedicate their efforts but as uncomfortable individuals who are subdued or – in the best of cases – treated with condescension. The psychiatrist figure appears as an example of the social fear of madness and difference in a practice depicted as hegemonic and homogenising that leaves no room for ‘alternative’ ways of seeing and being in the world. Psychiatry, in this conception, defines normality and curtails freedom. Ultimately, the songs under study oppose the power vested in psychiatry.

It was possible to identify an undercurrent of criticism of mental health practices, offering insights into the subculture's positions on the concept of mental disorder and expectations of its management. Among the criticisms, we found considerations of a reductionist approach heavily dependent on psychopharmacology and paternalistic approaches that do not consider patients’ opinions and do not respect their individuality. All this seems to reflect complaints about power distribution in the doctor–patient relationship, although in some cases, denying the pathological character of the mental disorder is added.

The depictions of the psychiatrist and treatments in punk songs are thought-provoking since, although the representations include distortions, stereotypes are rarely created out of thin air. The aesthetic amplifications, magnifications and falsifications in cultural representations have a potentially illuminating flip side if viewed from a broader perspective, since when artists describe psychiatric practices, they may actually be making a more global plea about the human condition and also a critique of some psychiatrists’ modes of practice.

## Supporting information

Pavez et al. supplementary material Pavez et al. supplementary material

## Data Availability

The data that support the findings of this study are available from the corresponding author, F.P., on reasonable request.

## About the authors

**Fabian Pavez** is a psychiatrist in the Mental Health Centre of Lorca, Murcia Health Service, Lorca, Spain. **Erika Saura** is a psychologist at the Association of Relatives and People with Mental Illness in Águilas and the Surrounding Area (AFEMAC), Aguilas, Spain. **Pedro Marset** is an Emeritus Professor in the History of Medicine Unit, Department of Social and Health Sciences, Faculty of Medicine, University of Murcia, Murcia, Spain.
