# Supplementary material for: Police of the psyche: the psychiatrist and psychiatry in Spanish punk songs
Source: BJPsych Bull. 2024 Jun;48(3):182–6. doi: 10.1192/bjb.2023.47 (PMC11134012; doi:10.1192/bjb.2023.47)
Supplement: Pavez et al. supplementary material [file S2056469423000475sup001.docx]

**Appendix: Bibliographic, documentary, and web sources**

Alfonso JA, Bocos A, Ramone E, Rins V, Aguilar O. To the end: 20 years of punk in Spain [Hasta el final: 20 años de punk en España]. Madrid: Libros Zona de Obras/SGAE; 2002.

Alfonso JA, Bocos A. I do not accept!!! 1980-1990: Ten years of Hardcore, Punk, Anger and Chaos [No Acepto!!! 1980-1990: Diez Años de Hardcore, Punk, Ira y Caos]. Aragon: Música Autónoma - Producciones Zambombo; 2007.

AA.VV. Dictionary of punk and hardcore (Spain and Latin America) [Diccionario de punk y hardcore (España y Latinoamérica)]. Madrid: Fundación Autor; 2011.

Grupos [Internet]. La Fonoteca. 2019 [cited 2019 Feb 5]. Available from: <http://lafonoteca.net/grupos/>

Foros [Internet]. Maneras de vivir. 2018 [cited 2019 Feb 5]. Available from: <http://www.manerasdevivir.com/foro/>

MusicaPunk.net [Internet]. 2013 [cited 2019 Feb 5]. Available from: http://musicapunk.net/
